# Clinical Impact of an Analytic Tool for Predicting the Fall Risk in Inpatients: Controlled Interrupted Time Series

**DOI:** 10.2196/26456

**Published:** 2021-11-25

**Authors:** Insook Cho, In sun Jin, Hyunchul Park, Patricia C Dykes

**Affiliations:** 1 Nursing Department College of Medicine Inha University Incheon Republic of Korea; 2 The Center for Patient Safety Research and Practice Division of General Internal Medicine Brigham and Women’s Hospital Boston, MA United States; 3 Department of Nursing National Health Insurance Service Ilsan Hospital Gyeonggi-do Republic of Korea; 4 Graduate School of Information & Telecommunications Konkuk University Seoul Republic of Korea; 5 Harvard Medical School Boston, MA United States

**Keywords:** clinical effectiveness, data analytics, event prediction, inpatient falls, process metrics

## Abstract

**Background:**

Patient falls are a common cause of harm in acute-care hospitals worldwide. They are a difficult, complex, and common problem requiring a great deal of nurses’ time, attention, and effort in practice. The recent rapid expansion of health care predictive analytic applications and the growing availability of electronic health record (EHR) data have resulted in the development of machine learning models that predict adverse events. However, the clinical impact of these models in terms of patient outcomes and clinicians’ responses is undetermined.

**Objective:**

The purpose of this study was to determine the impact of an electronic analytic tool for predicting fall risk on patient outcomes and nurses’ responses.

**Methods:**

A controlled interrupted time series (ITS) experiment was conducted in 12 medical-surgical nursing units at a public hospital between May 2017 and April 2019. In six of the units, the patients’ fall risk was assessed using the St. Thomas’ Risk Assessment Tool in Falling Elderly Inpatients (STRATIFY) system (control units), while in the other six, a predictive model for inpatient fall risks was implemented using routinely obtained data from the hospital’s EHR system (intervention units). The primary outcome was the rate of patient falls; secondary outcomes included the rate of falls with injury and analysis of process metrics (nursing interventions that are designed to mitigate the risk of fall).

**Results:**

During the study period, there were 42,476 admissions, of which 707 were for falls and 134 for fall injuries. Allowing for differences in the patients’ characteristics and baseline process metrics, the number of patients with falls differed between the control (n=382) and intervention (n=325) units. The mean fall rate increased from 1.95 to 2.11 in control units and decreased from 1.92 to 1.79 in intervention units. A separate ITS analysis revealed that the immediate reduction was 29.73% in the intervention group (*z*=–2.06, *P*=.039) and 16.58% in the control group (*z*=–1.28, *P*=.20), but there was no ongoing effect. The injury rate did not differ significantly between the two groups (0.42 vs 0.31, *z*=1.50, *P*=.134). Among the process metrics, the risk-targeted interventions increased significantly over time in the intervention group.

**Conclusions:**

This early-stage clinical evaluation revealed that implementation of an analytic tool for predicting fall risk may to contribute to an awareness of fall risk, leading to positive changes in nurses’ interventions over time.

**Trial Registration:**

Clinical Research Information Service (CRIS), Republic of Korea KCT0005286; https://cris.nih.go.kr/cris/search/detailSearch.do/16984

## Introduction

### Background

Inpatient falls are preventable adverse events that are the top 10 sentinel events in hospitals. Up to 1 million fall events occur annually in the United States, and the average cost of each event has been estimated at $7900–$17,099 (2019 USD) [1,2]. On average, ~400-700 falls occur annually in Korean tertiary academic hospitals [3-5].

Despite the availability of a considerable body of literature on fall prevention and reduction, falls remain a difficult, complex, and common problem that consume a great deal of time, attention, and mitigation efforts among nurses in practice [6,7]. Considering the studies on inpatient falls, most falls are preventable through tailored interventions and universal fall precautions [8]. However, fall prevention efforts are hindered by the inability to accurately estimate the risk of falling [9,10]. Several risk assessment tools developed using heuristic approaches have been widely used to estimate fall risk in practice. However, evidence regarding the efficacy of those tools is lacking [11,12], potentially resulting in a high false-positive rate and consequently increased burden on nurses. In addition, rating fall risk without identifying the underlying source uses nursing time but does not inform preventative interventions [13]. Our clinical observations reveal that nurses frequently tend to rely only on several universal precautions, not considering risk factors [14]. Implementation of cognitive, toileting-related, or sensory- and sleep-related assessments and interventions was rare.

The increased adoption of electronic health record (EHR) systems over the past decade has stimulated the development of predictive fall risk models using machine learning techniques, which are reported to exhibit better predictive performance than the existing fall risk assessment tools alone [15-18]. However, most of these models have not been validated in multiple settings, and their implementation is restricted by their use of aggregated data by hospital admission rather than by patient-days. None of these models have been evaluated prospectively to assess their performance or their impact on nursing practice. Nursing predictive analytics can include information regarding the likelihood of a future patient event through risk prediction models, which incorporate multiple predictor variables obtained automatically from the EHR. If such models are integrated into EHR systems, nurses can prospectively obtain information to inform their decision making on fall prevention intervention planning.

In this study, we used the prediction model that was developed in our previous study [18]. This model was designed to use nursing process data from EHRs and to consider nurses’ fall prevention workflow. Automatic and manual chart reviews were performed to identify all positive events in the retrospective data. The aim of this prospective study was to determine the effect of a predictive fall risk analytic tool on fall outcomes in patients admitted to 12 medical surgical units in South Korea, as well as their impact on nurses’ responses. This study hypothesized that providing nurses with information about patients’ likelihood of falling within 24 hours of admission, based on data routinely captured in EHRs, would enable nurses to provide risk-targeted interventions and contribute to a reduction in patient fall rates.

### Development of an Inpatient Fall Risk Prediction Model

This research team previously reported on the development of a fall risk prediction model [18]. Briefly, concepts of fall risk factors and preventive care were identified using two international practice guidelines [10,19] and two implementation guidelines [20,21] on preventing inpatient falls. Two standard vocabularies, the Logical Observation Identifiers Names and Codes [22] and the International Classification for Nursing Practice [22,23], were used to represent the concepts in the prediction model, which was then itself represented using a probabilistic Bayesian network.

The model was tested in two study cohorts obtained from two hospitals with different EHR systems and nursing vocabularies. The model concepts were mapped to local data elements of each EHR system, and two implementation models were developed for a proof-of-concept approach, followed by cross-site validation. The EHR data included in the model were demographics, administrative information, medications, Korean patient classification based on nursing needs, the fall risk assessment tool, and nursing fall risk prevention processes, including assessments and interventions. The two implementation models exhibited error rates of 11.7% and 4.87%, with *c* statistics of 0.96 and 0.99, respectively. The model performed 27% and 34% better than the existing Hendrich II tool [24] and the St. Thomas’ Risk Assessment Tool in Falling Elderly Inpatients (STRATIFY) system [25], respectively.

### Clinical Implementation of the Intelligent Nursing @ Safety Improvement Guide of Health information Technology System

The validation site model was implemented at a 900-bed public hospital in the metropolitan area of Seoul (Republic of Korea) that used STRATIFY to assess fall risks for all inpatients. The project, named Intelligent Nursing @ Safety Improvement Guide of Health information Technology (IN@SIGHT), was designed as a platform to support analytic tools as part of the infrastructure of a hospital EHR system, starting with a fall prediction analytic tool. The fall prediction analytic tool was integrated into the locally developed EHR system that had been in use for more than 10 years. The tool was deployed in 6 targeted nursing units (intervention group) on April 5, 2017, and all 204 nurses at those units automatically received the prediction results on a daily basis. This implementation process involved the chief of the Nursing Department, unit managers, unit champions, personnel of the Department of Medical Informatics, and the Patient Safety Committee. For 3 months before system deployment, three sessions of education on the IN@SIGHT system were provided to the intervention group, followed by peer-to-peer education provided by unit champions.

The Nursing Department decided to replace the existing STRATIFY with the analytic tool during this quasi-experimental study. The original model was customized by replacing the six data elements of STRATIFY with proxy data elements in the EHRs. The adjusted model, consisting of 40 nodes and 68 links, had an error rate of 9.3%, a spherical payoff of 0.92, and a *c* statistic of 0.87. Related work processes were redefined, and the existing fall prevention documentation screen of the EHRs was modified. The hospital decided to deliver the risk information in dichotomized format, with at-risk and no-risk categories at a cutoff point of 15%, which provided a high specificity of 89.4%. The analytic tool triggered an “at-risk” alert on the EHR system when the user selected an at-risk patient.

## Methods

### Study Framework and Objectives

**Figure 1:**
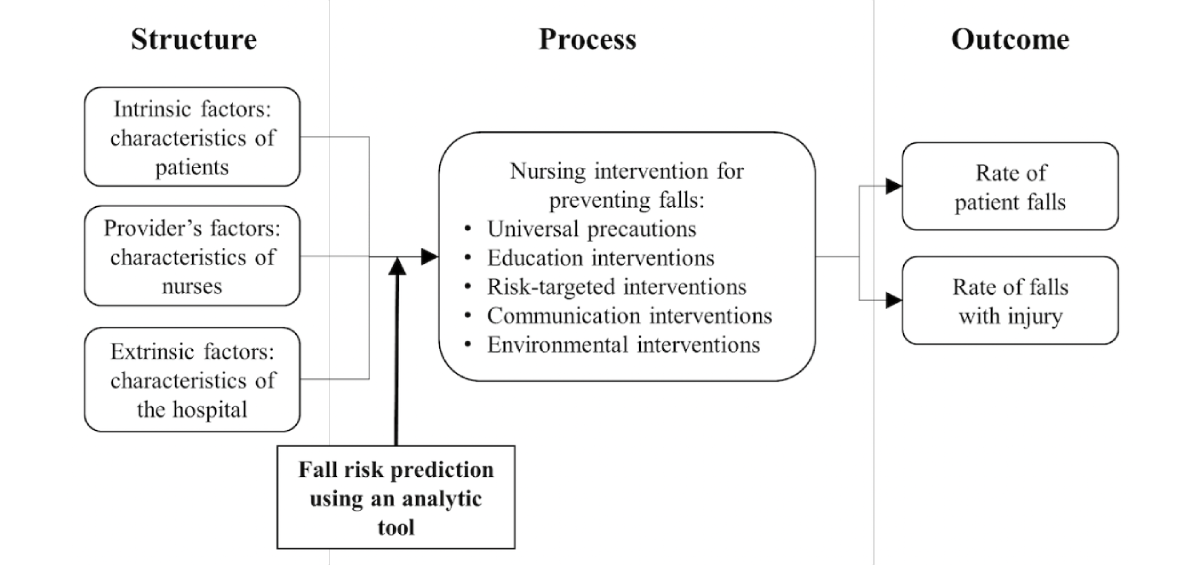
Conceptual framework of the study.

A study framework was developed based on a nursing role effectiveness model (Figure 1) [26]. The original model was based on the structure-process-outcome design of the Donabedian quality care model but was reformulated for this empirical testing, focusing on nurses’ independent roles in the process component. We assumed that the characteristics of the patients, nurses, and hospital were fixed because the study involved a single institution and the same medical-surgical units. The hypothesis being tested was that the intervention of fall risk prediction would affect the appropriateness of multifactorial interventions and would be followed by changes in outcome.

In accordance with the aim of this study, the impact of an electronic analytic tool for fall risk prediction on patient outcomes and nurses’ responses was explored by addressing the following specific research questions:

1. Did the predictive analytic tool influence the quality of nursing care as assessed using outcome indicators?
2. Did the predictive analytic tool affect nursing fall prevention activities provided to patients?
3. How did the effects change over time?

### Study Design and Setting

**Figure 2:**
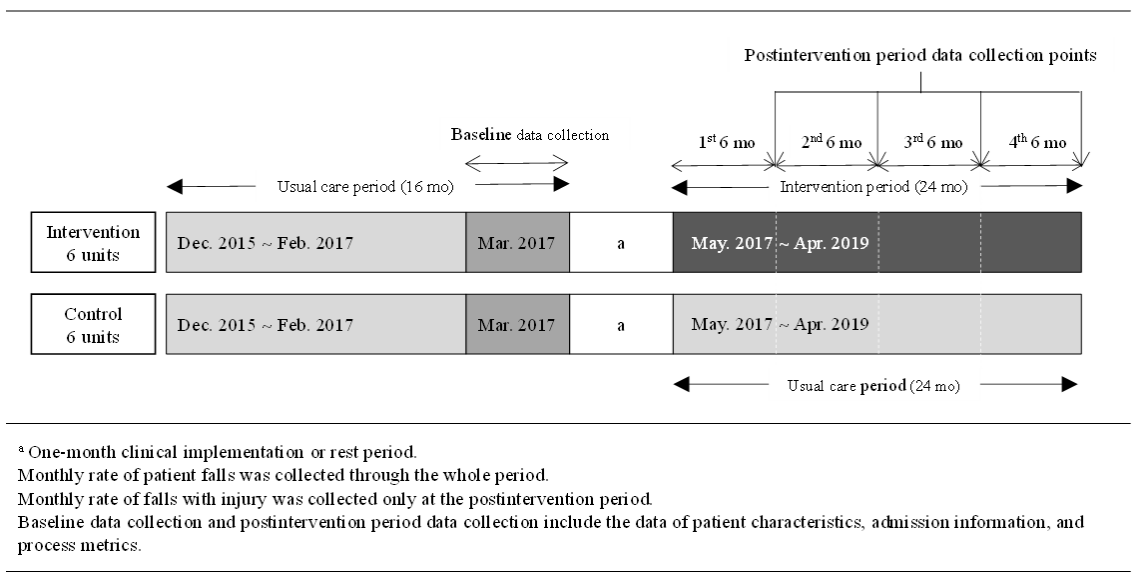
Nonequivalent-group design of the study.

This nonrandomized controlled trial used an interrupted time series (ITS) design. To control for bias due to time-varying confounders, such as other quality improvement (QI) initiatives occurring in parallel with the intervention and other events, the 12 medical-surgical units were selected and allocated to 1 of 2 groups using pairs of units matched according to the known fall rates and unit characteristics for individual units (Figure 2). All of the nurses and eligible patients participated in this study between May 1, 2017 and April 30, 2019. The patients met the following criteria: age ≥ 18 years and admitted to the hospital for >1 day in departments other than pediatrics, psychiatrics, obstetrics, and emergency care. The preintervention period was set at 16 months, which was the maximum retrospective time window. The 12 nursing units’ nurse staffing ratios were changed at the time due to a policy for comprehensive nursing service in the Korean government’s national health insurance. The postintervention period was 24 months. Process metrics, which measure the delivery of fall risk mitigation interventions by nurses to patients, were analyzed every 6 months.

This study was approved by the hospital’s ethical review board (IRB no. NHIMC 2016-08-005). A waiver of informed consent was granted by the IRB due to the QI nature of the intervention, thus enabling the inclusion of all patients and nurses in the participating units. This study followed the Transparent Reporting of Evaluations with Nonrandomized Designs (TREND) reporting guidelines [27].

### Intervention

Nurses in the intervention units received 24-hour fall risk prediction results for each patient every morning. These results could be overridden based on the nurses’ clinical judgment, such as when patients were receiving treatments, procedures, operations, or fall related high-risk drugs or whether they suffered a fall, seizure, or syncope. The fall risk predictions were created by the analytic tool using the data collected within the past 24 hours. For missing data, a priori values from the day before were assigned first, and then a replacement was used: a mean value for continuous variables and a modal value for categorical variables. Nurses in the intervention units used the STRATIFY risk assessment tool only on the day of admission. When an at-risk patient was selected by nurses in the EHR system, they received an alert once each shift informing them that the patient was at risk and were guided to a care plan screen that listed pertinent interventions ordered by priority according to the patient’s risk factors. Nurses in the control units used only STRATIFY to assess fall risk according to their individual clinical judgment. They were able to manually open the same care plan window through menu navigation but received no alerts for at-risk patients.

### Outcome Measures

The primary outcome was the overall rate of patient falls per 1000 patient-days during the study period, as defined by the National Database of Nursing Quality Indicator (NDNQI) outcome metrics of the American Nurses Association [28,29]:

A patient fall is a sudden, unintentional descent, with or without injury to the patient, that results in the patient coming to rest on the floor, on or against some other surface (e.g., a counter), on another person, or on an object (e.g., a trash can). NDNQI counts only falls that occur on an eligible inpatient unit that reports falls. When a patient rolls off a low bed onto a mat or is found on a surface where you would not expect to find a patient, this is considered a fall. If a patient who is attempting to stand or sit falls back onto a bed, chair, or commode, this is only counted as a fall if the patient is injured. All unassisted and assisted falls... are to be reported, including falls attributable to physiological factors such as fainting (known as physiological falls).

The secondary outcomes were the overall rate of falls with injury, and process metrics. The rate of falls with injury was also measured using the aforementioned NDNQI definition. Process metrics were defined according to the Institute for Healthcare Improvement definition as “process indicators that measure compliance with key components of evidence-based prevention” [30]. Methods for identifying and defining key components of fall prevention are described elsewhere [31]. In brief, nursing activities identified by international guidelines on preventing falls are categorized into 17 components; of these, 7 nursing intervention components were used in this study. Process metrics were used to determine whether nursing behaviors independently affected patient outcomes. Each process metric measured the proportion of at-risk patients who were provided with targeted interventions. For example, all hospitalized patients are expected to be assessed for fall risk factors within 24 hours of admission, and at-risk patients are expected to receive risk-targeted interventions within 24 hours of their risk designation.

### Data Collection

Monthly rates of patient falls were collected from 16 months before the experiment started (the preintervention period) from the hospital’s quality assurance department to provide a baseline reference for comparisons. However, monthly rates of falls with injury before the experiment were not comparable due to differences in the criteria used to calculate them; only severe injuries were used as a sentinel event at the hospital. For process metrics, 1 month of data from before the experiment were collected as a baseline. During the study, data on patient demographics and medications, nursing activities, STRATIFY data, and administrative information were collected from the EHR system, and fall data were collected from the hospital’s quality assurance department. To monitor and minimize the underreporting rate noted previously [31,32], the Nursing Department provided education to all units on the principles of reporting and documentation, and they provided monthly chart reviews and feedback.

### Sample Size and Statistical Analysis

The study hypothesis was that the fall rate would be reduced by 15% during the 24-month implementation of the prediction program. We conservatively estimated the required sample size based on previous research [18] by assuming a fall rate in the control group of 2.0 per 1000 patient-days, an average of 15,000 patient-days per unit over 12 months, and an average 1700 admissions. The required number of falls in the control group was calculated using a Poisson distribution: *D*_0_ = *z*^2^(*θ* + 1)/*θ*(log*_e_ θ*)^2^ [7]. We applied *z*=2.0; detecting a rate ratio (*θ*) of 0.85 between groups at the 5% significance level with a statistical power of 80% required 610 falls, which corresponded to a 24-month period for the 12 units.

The participant characteristics were compared using chi-square tests for categorical variables and *t* tests for continuous variables. The primary outcome of the rate of patient falls was compared by the controlled ITS, incorporating the control series analysis and the uncontrolled ITS [33]. We fit negative binomial models, including a lagged dependent variable to control for serial autocorrelation and monthly dummy variables, to generate seasonal fixed effects in each model. Each model included three variables to measure the relationship between time and patient fall rates: (1) a continuous variable to represent the underlying temporal trends, (2) a dummy variable for dates after May 1, 2017, to determine the change in fall rate related to the intervention, and (3) a continuous time variable beginning on that date to represent the change in slope. The coefficients of the second and third variables indicated whether the intervention had immediate and ongoing effects on the fall rate, respectively. The Student *t* test and a comparative time series analysis were conducted to analyze the rate of falls with injury, and chi-square analysis was used for the comparison of process metrics between groups.

## Results

### Patient Characteristics

This study involved 42,476 admissions of 40,345 unique patients in 12 units, corresponding to 362,805 patient-days in nursing units across both the control and intervention groups. In total, 2131 patients (5.02% of all admissions) were admitted to both an intervention and a control unit at different times. The patient characteristics differed significantly between the two groups (Table 1). Compared with the intervention units, the control units were characterized by older patients, a longer stay, fewer female patients, and more patients with a fall history at admission; rates of secondary diagnoses and surgical procedures were also higher. Approximately half of the patients in the intervention group had a respiratory or digestive disease or any form of cancer, while control patients had a greater diversity of primary diagnoses.

**Table 1:** Characteristics of patients in the intervention and control groups.

Variable			Intervention (n=24,336)		Control (n=18,140)		*P* value		
**Primary medical diagnosis, n (%)**									
	Respiratory or digestive disease	6150 (25.21)		3472 (19.14)		<0.001			
	Cancer	5990 (24.61)		2382 (13.13)		<0.001			
	Symptom or injury	2784 (11.44)		2561 (14.12)		<0.001			
	Cardiovascular disease	995 (4.09)		3096 (17.07)		<0.001			
	Benign tumor	860 (3.53)		211 (1.16)		<0.001			
	Infectious disease	514 (2.11)		388 (2.14)		<0.001			
	Neurologic disease	182 (0.75)		597 (3.29)		<0.001			
	Other^a^	6861 (28.19)		5433 (29.95)		<0.001			
**Other variables**									
	Age (years), mean (95% CI)	61.45 (61.23-61.67)		65.30 (65.05-65.54)		<0.001			
	Length of stay (days), mean (95% CI)	7.96 (7.91-8.00)		9.25 (9.13-9.37)		<0.001			
	Sex (female), n (%)	12,512 (51.41)		9053 (49.91)		0.002			
	History of fall at admission, n (%)	2873 (11.88)		4138 (23.58)		<0.001			
	Secondary diagnoses, n (%)	10,641 (43.73)		9361 (51.60)		<0.001			
	History of surgical procedures, n (%)	2483 (10.20)		8575 (47.27)		<0.001			

^a^Including genitourinary, musculoskeletal, eye, ear, and skin diseases.

### Primary Outcome: Rate of Patient Falls

There were 325 fall events in the intervention group and 382 in the control group. The mean monthly rate of falls decreased from 1.92 to 1.79 in the intervention group and increased from 1.95 to 2.11 in the control group**.** Controlled ITS analysis revealed that the postintervention versus preintervention change in the incidence rate ratio of the fall rate was −0.10 (SE 0.04, *P*=.014). There was no seasonal effect.

Due to the significant differences in patient characteristics between the control and intervention groups, we conducted separate before versus after comparisons between a period of time postintervention and the same period of time preintervention. In the intervention group, there was a significant reduction in the rate of falls of 29.73% (0.57 falls per 1000 patient-days) immediately postintervention (SE 0.14, *P*=.039). During the preintervention period, the slope exhibited a slightly decreasing trend (SE 0.08, *P*=.344), and after the intervention, the slope increased slightly but not significantly so (slope=0.01, SE 0.01, *P*=.059; Table 2). In the control group, there was a nonsignificant reduction in the rate of falls of 16.58% (0.16 falls per 1000 patient-days; SE 0.13, *P*=.20). The slope before the intervention increased (change in slope=0.08, SE 0.72, *P*=.292), while after the intervention, the slope increased slightly (change in slope=0.01, SE 0.01, *P*=.057).

**Table 2:** Results of interrupted time series analysis of rates of patient falls.

Group	Preintervention period trend	Change immediately after introduction of intervention	Postintervention period trend	
Intervention	−0.07 (−0.22 to 0.08)	−0.30 (−0.58 to –0.14)^a^	0.01 (<−0.01 to 0.02)	
Control	0.08 (−0.07 to 0.22)	−0.17 (−0.42 to 0.09)	0.01 (<−0.01 to 0.02)	

^a^*P*=.04.

Data are rate ratio (95% CI) values.

### Secondary Outcomes: Fall With Injury Rates and Process Metrics

During the intervention period, the mean monthly injury rate per 1000 patient-days was 0.42 in the intervention group and 0.31 in the control group. The comparative time series analysis revealed a nonsignificant increase in the rate ratio of 0.18 (*z*=1.50, *P*=.134).

Regarding process metrics, fall risk assessment was not conducted in almost three-quarters of patient-days in the control group, while in the intervention group, fall risk assessment was conducted on 100% of patient-days (Table 3). During the intervention period, the frequency of at-risk days was almost 40% in the control group but ranged from 24.5% to 34.6% in the intervention group. There was a high rate of implementation of a fall risk tool within 24 hours of hospital admission in both groups, although rates fluctuated over time in the control group. Rates of assessment of injury risk factors were assessed in all patients in the intervention group; these data were not available for the control group. Universal fall precautions and fall prevention education were provided to most patients in the control group consistently throughout the study period. Rates of implementation of communication and environmental interventions were initially significantly better in the control group than in the intervention group; however, those for the intervention group increased over time and had caught up with the control group by the third observation point. Although the rate of risk-targeted interventions incrementally increased in both groups, the intervention group showed better adherence than the control group at the fourth observation point (29.5% vs 18.1%, *P*<.001).

**Table 3:** Temporal changes in process metrics in the control and intervention groups.

Item			Baseline (1 month)		First 6 months of intervention		Second 6 months of intervention		Third 6 months of intervention		Fourth 6 months of intervention
**Base information**											
	Patient-days	8254 vs 4207^a^		45,133 vs 31,675		46,403 vs 39,733		44,418 vs 44,741		42,553 vs 43,161	
	Days on which no risk assessment performed, %	72.5 vs 73.4^b^		0 vs 72.6		0 vs 77.1		0 vs 71.7		0 vs 79.8	
	At-risk days, %	43.0 vs 42.1^b^		24.5 vs 43.5^c^		31.4 vs 38.6^c^		32.7 vs 42.9^c^		34.6 vs 41.5^c^	
**Process metrics: patients assessed within 24 hours of hospital admission, %**											
	Use of a fall risk tool	99.3 vs 98.6 ^b^		100.0 vs 99.2^c^		100.0 vs 70.8^c^		100.0 vs 95.3^c^		100.0 vs 98.8^c^	
	Injury risk factors (ABCs^d^)	0 vs 0^b^		100.0 vs 0		100.0 vs 0		100.0 vs 0		100.0 vs 0	
**Process metrics: at-risk patients who received within 24 hours of risk identification, %**											
	Universal precautions^e^	86.1 vs 100.0^c^		69.7 vs 78.9^c^		88.8 vs 99.9^c^		37.8 vs 99.9^c^		91.2 vs 99.9^c^	
	Education interventions^e^	86.1 vs 100.0^c^		69.7 vs 78.9^c^		88.8 vs 99.9^c^		33.1 vs 98.1^c^		79.6 vs 97.8^c^	
	Risk-targeted interventions	<0.01 vs <0.01		<0.01 vs <0.01		<0.01 vs <0.01		12.5 vs 13.3^b^		29.5 vs 18.1^c^	
	Communication interventions^e^	61.7 vs 79.4^c^		87.6 vs 99.9^c^		76.0 vs 81.1^c^		30.2 vs 38.7^c^		66.2 vs 66.7^b^	
	Environmental interventions^e^	61.7 vs 79.4^c^		87.6 vs 99.9^c^		76.0 vs 81.1^c^		39.5 vs 54.9^c^		76.7 vs 76.0^b^	

^a^All data shown as intervention group versus control group.

^b^Not significant.

^c^*P*<.001.

^d^ABCs: age, bone health, anticoagulants, and current surgery (function that was performed automatically in the intervention group).

^e^Data collection not categorized in detail from baseline to the second observation point.

**Figure 3:**
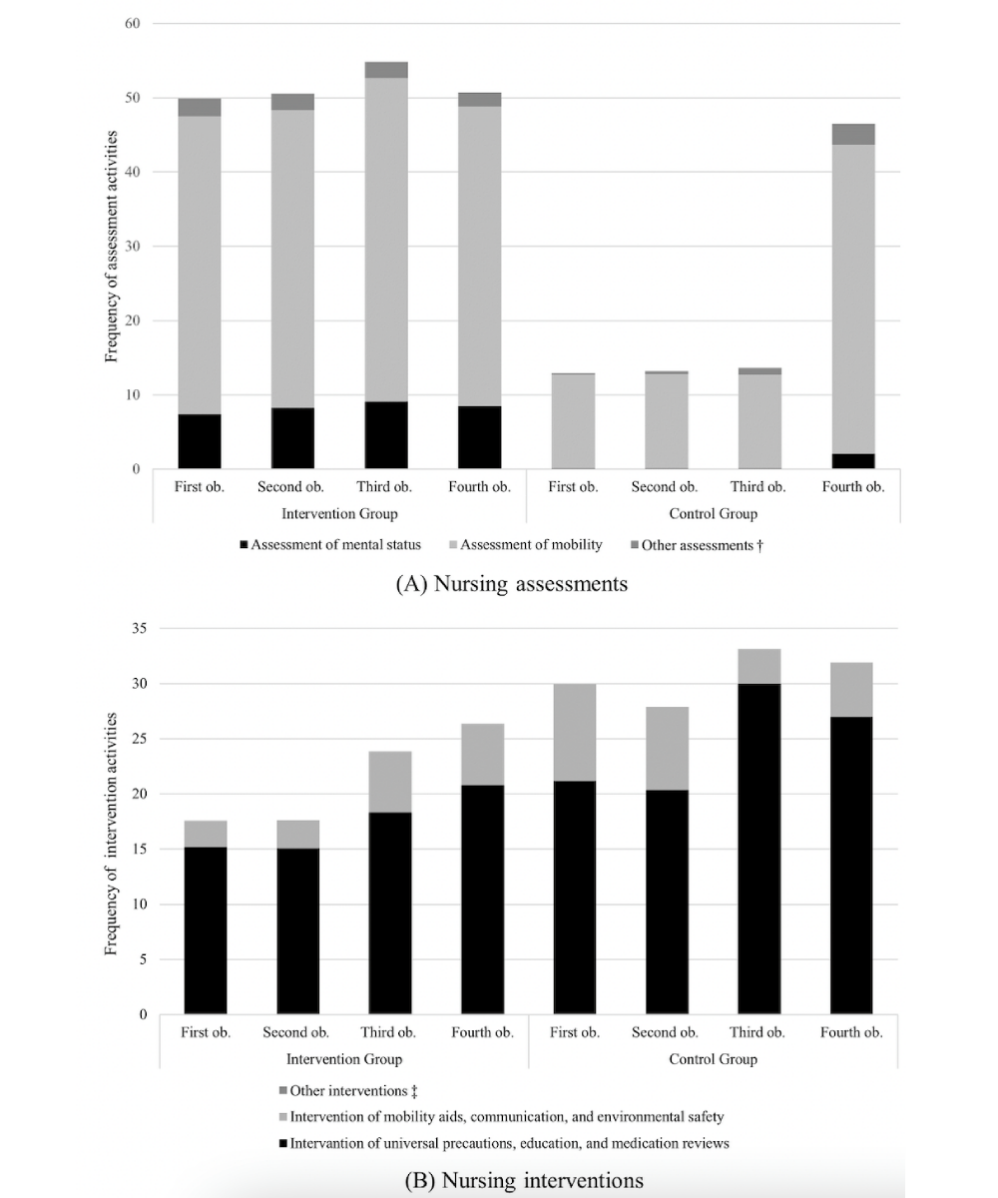
Changes in nursing assessments (A) and interventions (B) according to care components. ob.: observation point; †includes assessments of cognitive function, communication ability, gait status, incontinence, sleep pattern, and use of constraints; ‡includes interventions of toileting aids and for impaired mental and cognitive function, impaired sensory function, and sleep disturbance.

For the care components of nursing assessments, nurses in the intervention group performed various observation types, such as mental status, cognitive function, communication ability, and incontinence, including mobility, at each observation point (Figure 3A), while those in the control group appeared to focus largely on mobility assessments, the frequency of which suddenly increased at the last observation point. Universal precautions, education, and medication reviews were the most common interventions in both groups (Figure 3B). Although the frequency of interventions was lower in the intervention group than in the control group, there was a steady increase over time.

## Discussion

### Principal Findings

Implementation of an electronic analytic tool designed to predict fall risk was associated with reduced fall rates among inpatients at a public hospital in South Korea. However, comparison with the control group should be considered with caution due to notable differences in patient characteristics between the two groups. There was no significant difference in the rate of falls with injury between the control and intervention groups. Use of the electronic analytic tool was feasible, and it was accepted by nurses and improved the completion of risk assessments. Moreover, the process metrics for multifactorial and risk-targeted interventions for at-risk days were lower in the intervention group but increased over time. These findings suggest that although the effectiveness of an electronic analytic tool may be limited, it has potential as an aid to help nurses make informed clinical decisions.

The main challenges in this study were threefold: (1) random assignment of patients to the study groups was not possible; (2) it was not possible to control for co-interventions or external events at the hospital that may have affected the outcome, including QI activities; and (3) nurses’ understanding of the analytic tool developed by a machine learning approach was not assessed. These issues were managed by selecting only medical-surgical units and assigning patients according to the particular characteristics of each unit. A controlled ITS design was adopted to control for time-varying confounders. Finally, the development and validation process of the predictive model and the mechanism of chaining joint probabilities of a Bayesian network were introduced via user education sessions. However, during the study, the research team confronted additional issues that made interpretation of the results challenging. Discussion on these issues is valuable for future research into risk prediction and alerting in real-world settings.

The fall rates of 1.79 and 2.11 in the intervention and control groups, respectively, in this study were lower than previously reported rates of 2.08-4.18 for an intervention study involving a cluster randomized controlled trial (RCT) in four urban US hospitals [34], 3.05 for a cluster RCT in Australia [7], and 2.80 for a US intervention study [35]. However, differences in the patient populations and in the structural elements at the facilities preclude direct comparison [36]. The low fall incidence rate in this study allowed us to observe changes in nursing behaviors over a 24-month follow-up period. A fall prevention intervention will not be effective if it does not influence nurse behaviors. We focused on how the analytic tool can influence nursing behaviors in order to ensure that interventions that are beneficial to patients are routinely provided. Our findings revealed that the intervention group performed more multifactorial patient assessments than the control group; however, the interventions in both groups were limited. Most of the preventive components involved education and medication review, which is perhaps unsurprising since these precautions are routinely applied to all inpatients regardless of their fall risk. Interventions associated with toileting, impaired mental and cognitive function, impaired sensory function, and sleep disturbance were rarely observed in both groups.

According to international guidelines for preventing falls [10,19-21], multifactorial assessment of risks and multifactorial, risk-targeted interventions are basic components of fall prevention strategies. Application of the analytic tool in this study ensured that risk factors were monitored daily for each patient in the intervention group and that alerts were delivered to their nurses via the hospital EHR system. A large increase in data-seeking and data-gathering activities was observed during the first 6 months of observation, whereas notable increases in overall interventions and risk-targeted interventions appeared 12 and 18 months later, respectively. This suggests that adoption of this new approach and its processes by nurses was time dependent and stepwise, in line with the findings of surveys conducted repeatedly during the study period [37]. Those surveys revealed that some nurses reported neutral or even slightly negative attitudes and experiences at the beginning of the study. However, the proportion of negative responses gradually decreased over time. These findings can be understood in terms of the non-adoption, abandonment, scale-up, spread, and sustainability (NASSS) framework [38] to explain the success of technology-supported health or social care programs. Staff members are often initially more concerned about threats to their scope of practice or to the safety and welfare of patients, leading them to initially gather more information about risks. A previous qualitative exploration study [39] that used one-on-one and focus group interviews to investigate nurses’ perception of predictive information and how they act upon it found that nurses attempt to gather more information from other sources and review more detailed predictions during periods of uncertainty. Time delays in adoption and changing of behaviors are expected, given that predictive information is relatively new to nurses. The other relevant domain of the NASSS framework is the readiness of a hospital for a predictive analytic tool. The understanding and support, antecedent conditions, and level of readiness for a novel tool at the board level might influence the uptake time by nurses and the internal drivers for scaling up the tool.

### Study Limitations

This study had limitations. The control group patients had more comorbidities that rendered them more vulnerable to falls than the intervention group. They were on average 4 years older, had a hospital stay that was 1.3 days longer, and had a greater history of falls. These variables are known important covariates [19], and we did not balance these covariates in the ITS experiment. The differences in these covariates between the two study groups may be attributable to an ascertainment bias issue; it is possible that rather than there being a true reduction in fall rates in the intervention group, more patients at a lower risk of falls were included in that group. Evaluation of the baseline data suggests that the nurses in the control group delivered significantly more fall-preventive interventions to their patients than did those in the intervention group, including more additional risk assessments, universal precautions, educational interventions, and communication and environmental interventions. Thus, control group patients were both more likely to fall and to receive more fall-preventive interventions from nurses. It is unclear how these counterbalancing factors interact and how they may have impacted the outcomes of this study; however, it can be assumed that the greater provision of interventions appears to have contributed to the reduced fall risk in the control group.

The temporal changes in process metrics and nursing activities can provide important clues as to the overall impact of this trial. In a previous study [18], we found that the analytic tool predicted about 20% of patient at-risk days, which was about a half of the rate classified using STRATIFY (~40%-50% of patient-days as at-risk days). The actual rate of falls in the hospital was much lower, at around 0.2% of patient-days. We assumed that more precise up-to-date predictions of fall events would decrease the nurses’ burden on redundant interventions induced by false-positive warnings from STRATIFY. The analytic tool approach did not affect the universal fall precautions, but risk-targeted interventions, education, communication, and environmental interventions significantly increased compared with the control group, which remained at a steady state. These findings are meaningful, given that multifactorial interventions, including risk-targeted interventions, prevent anticipated physiologic falls, which are responsible for more than 70% of inpatient falls [34,40]. These process metrics revealed slow but explicit changes in nursing interventions, which indicates that the processes underlying care elements had changed and we could expect subsequent improvement in patient outcomes [41]. Continuous measurement and analysis of process metrics informed our understanding of the effects of interventions on patient outcomes and our interpretation of the effects of confounding, which has rarely been accounted for in previous studies [7,34,42].

### Study Design Limitations

The design of this study had several limitations that impacted the interpretation of its findings. First, due to the unexpected differences in baseline characteristics between the intervention and control groups, robust conclusions could not be drawn regarding comparison of the primary outcome between them. Future studies should implement matching techniques, such as propensity score matching [43] or synthetic control approaches [44], to ensure balance between known covariates. Second, implementation of the intervention at a single site over a long study period introduced several challenges that could have reduced the effects of this study trial. One challenge was an unexpected event at the hospital whereby one nursing unit in each group moved to a new location 1 month after study initiation, and nurse staffing was thus reorganized due to the physical reconstruction of the hospital buildings. The fall rate markedly increased for several months in that intervention unit compared with the other five units in the group. However, the control group unit that was relocated showed only a slight increase compared with the other units in their group. The relocations were accompanied by changes in staff nurses and in the medical diagnoses of patients, both of which may have increased the burden on nurses and induced the sudden increase in the fall rate at the unit. Another unexpected event was the routinization of hourly nursing rounds to all inpatients mandated by the hospital’s safety committee during the final intervention period. This may have accounted for the sudden increase in nursing assessments observed in the control group. In addition, conducting this study at a single hospital may have an indirect effect on the control units. The unit managers of the control group were also involved in the QI initiatives of this study, along with those of the intervention units. This could have caused a contamination effect, whereby the managers of the control group learned about the study intervention and decided to adopt it for their own units. Third, we were unable to compare the injury fall rates between the pre- and postintervention periods; therefore, the impact of the analytic tool on the rate of falls with injury remains unknown.

Inpatient fall prevention is a difficult and complex issue, for which there is little high-quality evidence [7,45]. Even after taking into account the study limitations, the findings of this early-stage evaluation of an analytic tool demonstrated that the interaction between the tool and nurses was adequate and the tool may have influenced nurses’ decisions on preventive interventions. The analytic tool developed herein represents a potential new approach for patient-level risk surveillance and for improving the efficacy of interventions at the system level. The findings and challenges discussed herein will contribute to improving further research on risk prediction and alerting in real-world settings.

### Conclusions

This was an early-stage clinical evaluation of a nursing predictive analytic application designed to forecast patient fall events in real time and at the point of care to improve outcomes and reduce costs. The effectiveness of the electronic analytic tool was supported only by the before-after comparison, not by the intervention-control comparison. Nurses were amenable to using the tool in practice, and over the course of the study, there were meaningful changes in process metrics, leading to more multifactorial and risk-targeted interventions to prevent patient falls.

## Abbreviations

EHR: electronic health record
IN@SIGHT: Intelligent Nursing @ Safety Improvement Guide of Health information Technology
ITS: interrupted time series
NASSS: non-adoption, abandonment, scale-up, spread, and sustainability
NDNQI: National Database of Nursing Quality Indicator
QI: quality improvement
STRATIFY: St. Thomas’ Risk Assessment Tool in Falling Elderly Inpatients
TREND: Transparent Reporting of Evaluations with Nonrandomized Designs
